# Thematic series on emerging leaders in biological engineering: convergence and new directions

**DOI:** 10.1186/s13036-019-0146-7

**Published:** 2019-02-21

**Authors:** Raj R. Rao

**Affiliations:** Department of Biomedical Engineering, College of Engineering, University of Arkansas, Fayetteville, AR 72701 USA

## Abstract

Over the past two decades, the biological engineering research community has increased its efforts to promote ‘Convergence’. Many research endeavors have thus involved the synergy of multiple perspectives and approaches from originally distinct fields towards driving creative innovative solutions to address many grand challenges that face our society.

Here, we introduce the JBE thematic series on emerging leaders in biological engineering. The aim of the series is to disseminate the latest developments in research that integrate the principles of biology and engineering methodologies to address grand challenges in human health and the health of the planet. This thematic series highlights exciting new areas and emerging leaders who are addressing many of these challenges.

## Introduction

This editorial will highlight the emerging themes and leaders linked to biological engineering research. Broadly, the topical areas that JBE promotes are:

- Synthetic biology and cellular design
- Biomolecular, cellular and tissue engineering
- Bioproduction and metabolic engineering
- Biosensors
- Ecological and environmental engineering
- Biological engineering education and the biodesign process

Since ‘Convergence’ is an integral part of biological engineering research [1], there is a demand for a forum in which the scientific community can be made aware of cutting-edge developments that address many grand challenges.

As emphasized by Dr. Jeong-Yeol Yoon in an editorial published in 2017 to highlight the 10th year of JBE, biological engineering is not only unique in its inclusive nature, but rather exhibits its exceptional potential to connect different engineering disciplines or even to connect other applied science disciplines to converge into a whole new research theme, technology, or discipline [2]. Specifically, a system-wide approach typically adopted in biological engineering, often converges entire systems, making the biological engineering discipline substantially broad-based and inter−/multidisciplinary.

In the context of future of the field and in conjunction with multiple grand challenges addressed by the U.S. National Academy of Engineering [3], Dr. Yoon highlighted specific examples of emerging inter−/multi-disciplinary biological engineering to include synthetic biology, personalized medicine, big-data in environmental engineering, field-ready and handheld biosensors, and sustainability in ecological systems. Although these are listed as unique examples, there are many exciting themes that emerge and provide new opportunities that may lie under the broad umbrella of biological engineering, where JBE provides an exciting forum to promote.

## The JBE thematic series on “Emerging leaders in biological engineering”

The JBE thematic series on ‘Emerging leaders in biological engineering’ [4] aims to highlight exciting new areas and emerging leaders who are addressing many of these disparate challenges. This series contains many articles that were considered highly significant by members of the editorial board and the validation of multiple reviewers who have been involved in the peer review process. For the purpose of this series, authors have extended their prior contributions and proposed new directions in their research programs. For this initial offering of the thematic series, we have highlighted articles that aim to capture the breadth and impact of biological engineering. Broadly, topics are related to personalized medicine, engineering systems for understanding human development and diseases, cross-species relationships, cell-material interactions, biomarkers, drug delivery and imaging biological systems. This is intended not only for a wide audience of researchers but to highlight emerging research topics and researchers who are spearheading innovative research programs, that highlight the convergence across disciplines.

## Concluding remarks

As summarized in this editorial, JBE is taking the lead on promoting research articles that operate at the interface of multiple disciplines with the goal towards promoting convergence of ideas, concepts and perspectives. The range of articles that JBE publishes is broad-based and inter−/multidisciplinary. In the future, we aim to establish a call for the ‘emerging leaders’ thematic series every two years, so that the research community is made aware of the evolving topics and novel applications. We aim to make the thematic series take a central role in the exploration of innovative research topics and to work closely with the community to fulfill this aim. We invite the members of the research community to express ideas and provide suggestions for continuous discussions.

## Abbreviation

JBE: Journal of Biological Engineering
